# Epigenetic Therapies in Melanoma—Targeting DNA Methylation and Histone Modification

**DOI:** 10.3390/biomedicines13051188

**Published:** 2025-05-13

**Authors:** Adrian Bogdan Tigu, Andrei Ivancuta, Andrada Uhl, Alexandru Cristian Sabo, Madalina Nistor, Ximena-Maria Mureșan, Diana Cenariu, Tanase Timis, Doru Diculescu, Diana Gulei

**Affiliations:** 1 Department of Personalized Medicine and Rare Diseases, MEDFUTURE—Institute for Biomedical Research, “Iuliu Hațieganu” University of Medicine and Pharmacy, 400349 Cluj-Napoca, Romania; 2 2nd Department of Obstetrics and Gynecology, “Iuliu Hațieganu” University of Medicine and Pharmacy, 400006 Cluj-Napoca, Romania

**Keywords:** melanoma, DNMT, histone, methylation, epigenetic mechanisms

## Abstract

Skin cancer prevalence has increased during the last decades, with the last years serving as a pivotal moment for comprehending its epidemiological patterns and its impact on public health. Melanoma is one of the most frequently occurring malignancies, arising from a complex interplay of genetic factors, environmental factors, lifestyle and socio-economic conditions. Epigenetic changes play a critical role in tumor development, influencing progression and aggressiveness. Epigenetic therapies could represent novel therapeutic options, while drug repositioning may serve as a viable strategy for cancer treatment. Demethylating agents, commonly used in hematological malignancies, show promising results on solid tumors, including melanoma. Methylation patterns are responsible for tumor development by modulating gene expression, while histone acetylation influences DNA processes such as transcription, replication, repair, and recombination. This review aims to identify existing potential therapeutical approaches using therapeutic agents that can modulate DNA methylation and histone modification, which can lead to tumor inhibition, cell death initiation and reactivation of tumor suppressor genes.

## 1. Introduction

Skin cancer represents an important public health issue, being one of the most common types of cancer and it is caused by a complex relationship between genetic factors, environmental factors, behavior and socio-economic factors [1]. According to Siegel et al., the incidence of melanoma has been rising steadily since 1975, with over 100,000 new cases projected in the United States of America for 2024 [2]. One out of five cases of skin malignancies is confirmed to be melanoma.

Melanomas arise from melanocytes—cells derived from the neural crest—and can develop not only in the skin and eyes but also in other tissues [3,4]. Cutaneous melanoma is the most common and includes several subtypes: superficial spreading melanoma–characterized by slow horizontal growth before invading deep layers; Nodular melanoma–growing vertically, aggressive and in deeper layers; lentigo maligna melanoma–frequent in the elderly population, slow growing at the beginning and later becoming invasive; acral lentiginous melanoma–frequently appears on the palms, under the nails and more common in darker skin population; and desmoplastic melanomas–highly invasive, rare, and mostly in sun-exposed areas. The second type of melanoma based on localization and characteristics is mucosal melanoma, which arises from cells in mucous membranes. This is an aggressive form and is often diagnosed late. The third type is ocular melanoma, also named uveal or choroidal melanoma, which develops in the eye, it is less common but can generate metastatic sites [5,6]. Other rare types of melanomas are marked in the literature–nevoid melanomas that develop from benign moles, spitzoid melanoma that is hard to diagnose due to the similarities with benign Spitz nevi, or amelanotic melanoma which has no pigmentation [7,8,9].

In 1966, Clark introduced a method for evaluating melanoma based on the depth of invasion into the dermis and subcutaneous tissue. By dividing the skin into histological compartments, he observed that the risk of metastasis increased as melanoma cells penetrated deeper through each layer. The scoring was named Clark levels and included five different levels: level one with melanoma in situ (in the epidermis); level two with the invasion of single cells into the papillary dermis; level three with many melanoma cells detected in the papillary dermis; level four where the invasion reaches the reticular dermis; and level five with subcutaneous fat invaded by melanoma cells [10,11].

Later, in 1970, Breslow’s classification suggested a stratification based on the depth of the invasion: stage I with no more than 0.75 mm; stage II down to 1.5 mm; stage III reaching 2.25 mm; stage IV between 2.26 and 3 mm; and stage V characterized by invasion greater than 3 mm [11,12].

Nowadays, the Tumor-node-metastasis system (TNM) is used for staging patients diagnosed with melanoma, combining the histologic features of the primary tumor, the lymph node disease and the distant metastasis. The TNM system is used in conjunction with Breslow’s thickness and Clark’s level of invasion to provide a more precise staging of melanoma, which can improve prognostic accuracy and potentially enhance patient survival outcomes [11,13,14].

The World Health Organization proposes a classification based on UV exposure, as the UV radiation effect is linked to almost 75% of all newly diagnosed melanoma cases. Long exposure to UV radiation increases the chance of developing melanoma, with the melanocytes developing genetic lesions [6,15,16]. The Low UV Cumulative Sun Damage (CSD) includes superficial spreading melanomas (category I), with mutations in *BRAFV*600, *CDKN2A*, *TER*, *PTEN*, *TP53* and *NRAS*. The High UV CSD includes two melanoma subtypes: Lentigo maligna melanoma (Category II), with mutations in *NRAS*, *BRAF-nonV600E*, *KIT*, *TERT*, *CDKN2A*, *PTEN* or *TP53;* and Desmoplastic melanomas (Category III) with mutations in *NRAS*, *PIK3CA*, *NFKBIE* or *NF1*. Other six categories of melanoma are marked as low or non-UV CSD, and display several mutations or rearrangements in different genes, as follows: category IV–Spitz melanoma with mutations in *CDKN2A* and *HRAS*, and rearrangements in *ALK*, *NTRK1* and *NRTK3*; category V–acral melanoma with mutations in *KIT*, *NRAS*, *BRAF*, *TERT* and *CDKN2A* and rearrangement in *ALK*, *NRTK3*; category VI–mucosal melanoma with mutations in *KIT*, *NRAS*, *BRAF*, *SF3B1*, *CDK4*, *CDKN2A*, and *CCND1* and *MDM2* amplification; category VII–melanoma in congenital nevus that displays mutations in *NRAS* and *BRAFV600E*; category VIII melanoma in blue nevus with mutations in *GNA11*, *BAP1*, *EIFAX*, *SF3B1*, *GNAQ* and *CYSTALTR2* genes; and category IX–uveal melanoma that displays mutations in *GNA11*, *GNAQ*, *BAP1*, *EFIAX*, *SF3B1*, *CYSLTR2* and *PLCB4* [6].

**Figure 1:**
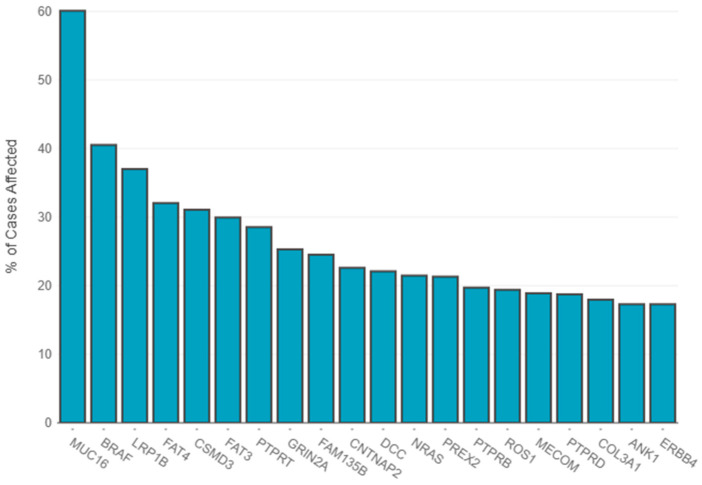
Distribution of most frequently mutated genes. The primary diagnosis filter included amelanotic melanoma (7 cases), epithelioid cell melanoma (20 cases), malignant melanoma–no other specified (nos) (513 cases), melanoma–nos (640 cases), mixed epithelioid and spindle cell melanoma (38 cases), nodular melanoma (21 cases), spindle cell melanoma–nos (23 cases), spindle cell melanoma, type b (9 cases) and superficial spreading melanoma (6 cases). The statistics were generated on 16 February 2025—https://portal.gdc.cancer.gov.

According to TCGA, several gene mutations were detected in melanoma patients with different incidence rates, with *MUC16*, *BRAF* and *LRP1B* the top three most frequent mutated genes, as depicted in Figure 1.

Despite the multiple staging systems, and the discovery of novel targeted therapies in melanoma, the disease remains one of the most frequent diagnoses in high-income countries. Furthermore, exposure to UV radiation contributes to genetic abnormalities that sustain the development of malignant cells [17,18].

Alternative therapeutic strategies offer promising avenues for advancing melanoma management and improving patient outcomes. Epigenetic modulation could influence melanoma cell development and aggressiveness; thus, the use of epigenetic therapies may offer alternative therapeutic strategies. Drug repositioning might be an alternative for melanoma therapy, as in other types of solid tumors studies reveal that the use of demethylating agents that were initially used for hematological malignancies could represent a successful approach [19,20,21,22]. Furthermore, Conway et al. classified stage 2 and 3 primary melanomas based on DNA methylation patterns thus proving stratified epigenetic subclasses with certain clinical and prognostic significance [23]. In rarer conjunctival melanoma cases, it was revealed as well that those methylation changes could serve as biomarkers for diagnosis [24].

Epigenetic modifications, especially DNA methylation and histone alterations, play a key role in melanoma progression, inducing changes in gene expression without affecting the DNA sequence. Patterns of aberrant DNA methylation, hypermethylation of the promoters of tumor suppressor genes and widespread hypomethylation, mainly play a role in malignant amplification. Other histone modifications, such as acetylation and methylation, affect the architecture of chromatin and the transcription process. Currently, attention is directed toward identifying inhibitors of DNA methylation and histone-modifying enzymes with a view to restoring normal gene expression and limiting tumor growth. This review aims to highlight the epigenetic therapies that may be used toward treating melanoma by modulating DNA methylation patterns or histone modifications and targeting the key regulators of these epigenetic mechanisms.

## 2. DNA Methylation and Its Role in Melanoma

DNA methylation is an important epigenetic event that involves chemical modifications in the DNA. It is a reaction catalyzed by DNA methyltransferases (DNMTs), enzymes that transfer the methyl (CH_3_^−^) from S-adenyl methionine (SAM) to the cytosine on the fifth carbon, and the resulting molecule is 5-methyl cytosine (5-mC). DNMTs have different roles in the methylation mechanism: the de novo DNMTs (DNMT3a and 3b) determine the methylation pattern, while DNMT1 is involved in the DNA replication, creating a copy of the methylation pattern [25].

The regions rich in cytosine and guanine (CpG islands) have more CpG than the rest of the genome, and most of the islands are located in the promotor regions of the genes. The methylation of the CpG islands in the promoter region is associated with gene silencing; thus the methylation and demethylation of these regions can modulate gene expression in development and differentiation [26,27].

Hypermethylation and hypomethylation can both be involved in carcinogenesis and can sustain tumor cell survival and proliferation. The global hypomethylation in cancer DNA and an increased methylation in transcription regions can influence tumor development. Hypermethylated regions of tumor suppressor genes and global hypomethylation can stimulate tumor growth; thus, understanding the mechanism of DNA methylation can provide insights into tumor development and may offer a new perspective for therapeutic strategies that can modulate this mechanism. It is well known that hypomethylation in tumor cells is related to protooncogene overexpression and overproduction of growth factors that maintain uncontrolled cell growth. Moreover, the DNA methylation pattern can be used to discriminate between malign and benign cells, with high potential as a diagnosis tool [28,29,30,31,32].

According to Fu et al. [33], the abnormal levels of 5-mC can modulate gene expression in melanoma, as in other malignancies. Global DNA hypomethylation could promote tumor cell proliferation by supporting their survival; also, hypomethylating the repetitive sequences could induce chromosomal instability [34,35]. Furthermore, the hypermethylation of different tumor suppressor genes can disrupt key biological processes such as DNA repair mechanism, apoptosis, cell cycle and transcription [33]. It was proven that there is an association between increased metastasis capacity and hypermethylation of several genes such as *LINE-1*, *KIT*, *TNF*, *MITF* and *CDKN2A* [36,37,38,39]. Cell cycle arrest was observed in malignant melanoma cells with hypermethylated *MMP-9*, *ASC/PYCARDC* or *TPM1* [40,41,42,43]. Other key processes such as cell-to-cell or cell-to-matrix adhesion, actin-mediated motility or proliferation were stimulated by the hypermethylation of different genes that control these pathways [33,44].

**Figure 2:**
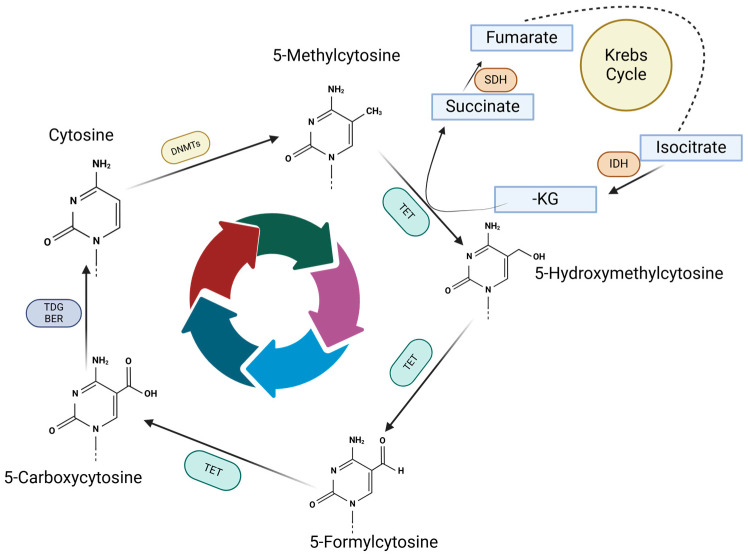
Methylation and demethylation cycle. Methyl group is attached to cytosine by DNMTs, which is further converted into 5-Hydroxymethylcytosine by TET enzymes. Further, TET enzymes start the demethylation process by generating 5-Formylcytosine and 5-Carboxycytosine. 5-Carboxycytosine could become a cytosine under TDG/BER action removing the residues on carbon 5, which will be available for another methylation.

The cycle of changes in DNA methylation controls the silencing and expression of different genes by the fluctuation between methylated and demethylated cytosines. As depicted in Figure 2, cytosine methylation is reversible and the process is modulated by DNMTs and TET enzymes, which facilitate the methyl addition to cytosine and then generate the intermediate cytosine compounds and finally the demethylated cytosine. TET enzymes require Krebs cycle metabolites, mostly ketoglutarate (-KG) which is essential for TET activity. Active DNA demethylation is dependent on iron (Fe^2+^) and oxygen; thus, Isocitrate Dehydrogenase (IDH) plays a critical role in epigenetic processes via its metabolic activity, succinate accumulation and metabolic shifts.

### 2.1. DNA Methylation, Tumor Suppressor Gene Silencing and miRNA Genes in Melanoma

Carcinogenesis can be driven both by epigenetic and genetic events. In case of genetic events, the DNA sequence is affected, and the final gene product might display defects. In the case of epigenetic events, the changes are reversible; thus, the changes in DNA, RNA or histones can be targeted with different therapies, in order to reverse gene silencing or overexpression. Gene silencing is controlled by molecular tags that mark if the gene should not be translated. In some cases, these tags may be added aberrantly and cause the silencing of genes that prevent tumor development. DNA methylation is using the tagging system for silencing or stimulating gene expression.

**Tumor suppressor genes in melanoma and other malignancies.** In other malignancies, as well as in melanoma, methylated promoters of tumor suppressor genes trigger gene silencing and further promote the initiation and development of cancer [45,46]. *APC*, a tumor suppressor gene involved in proliferation, migration, DNA repair and chromosomal segregation, was identified as silenced in different digestive cancers and hepatic cancer [47,48]; *BRCA1* and *RAD51C*, both involved in DNA repair, were silenced in ovarian, breast and gastric cancers [49,50,51,52,53]; *MLH1* and *MSH2*, two genes involved in mismatch DNA repair, were silenced in colon, gastric, lung and endometrial carcinomas; moreover, their dysfunction led to microsatellite instability [54,55,56,57,58].

In melanoma, focal DNA hypermethylation of several tumor suppressor gene promoters was highlighted by Micevic et al. Indeed, *RASSF1A*, *PTEN* and *p16/14* silencing was associated with *NRAS* mutations, decreased gene transcription, loss of expression and dysregulated biological functions, such as cell cycle and DNA repair [59].

Different methylation patterns affect genes that control critical pathways for cancer cell survival, proliferation, apoptosis, cell cycle, interaction with the immune system or metabolism. Promoters of *COL1A2*, *HSPB6*, *NPM2*, *MT1G* or *DDIT4L* were identified as hypermethylated in melanoma cells suggesting that they can be used as predictors for melanoma progression [59,60,61].

Conway et al. proposed a panel of hypermethylated and hypomethylated genes that showed differences in terms of methylation when comparing melanocytic nevi and melanoma cells [61].

**miRNA genes.** Methylation of miRNA genes is associated with the survival, proliferation, and migration of melanoma cells and regulates the EMT signaling cascade, as well as major pathways such as p53 and PI3K. For example, miR-34a, recognized as a p53 target and a tumor suppressor, is frequently silenced by methylation in melanoma. Although liposomal miR-34a mimic showed potential to cure acral melanoma, its use was abolished due to major immunological side effects. MiR-34b and MiR-34c regulate ULBP2, MET, CDKs, and N-myc and, through changes in cytoskeletal dynamics, they exert reduced invasion and adhesion [62,63,64]. MiR-211, which is regulated by DNMT1, prolongs the life of HIF-1α and inhibits EMT through RAB22A. MiR-203 inhibits invasion by targeting BMI1, whereas miR-375 is known to be hypomethylated and correlated with melanoma progression. Re-expressing miR-375 inhibits proliferation and motility in melanoma cells. Conversely, silencing miR-18b leads to an increase in MDM2, which suppresses p53. Hypomethylation would thus reveal various miRNAs, including miR-34b/c, miR-489, miR-375, miR-132, miR-519b, and miR-200a, as alternate targets of therapeutic opportunity. MiR-205 is commonly silenced in melanoma and inhibits migration by modulating ZEB2 and E-cadherin. Methylation is also responsible for regulating a cluster of miRNAs found at 14q32, such as miR-376a/c, which suppresses growth and migration by downregulating IGF1R. Moreover, miR-31, which gets silenced in melanoma, suppresses migration and invasion by targeting SRC, MET, RAB27a, and MAP3K14 [59,65,66]. DNA methylation is critical for miRNA regulation, as DNA methylation of genes encoding tumor-suppressing miRNA promotes tumorigenesis, therefore hypomethylation of these regions could have therapeutic potential [67].

DNA methylation coexists in a tight relationship with histone modifications. For instance, PRC2 mediates H3K27me3 to silence tumor suppressor genes. In melanoma, such a combination is relevant, since it enables cell survival, undifferentiation, and resistance to therapy [68].

In conclusion, hypermethylation in miRNA genes and tumor suppressor genes can potentiate tumor development and increase the risk of therapy resistance or relapse, making the hypomethylation of these regions a potential target for improved therapies.

### 2.2. Demethylating Agents: DNMT Inhibitors

As DNA methylation was confirmed as a key biological process in gene expression and silencing, various epigenetic modulators have been under investigation during the last decades, showing that targeting DNA hypermethylation or hypomethylation may be a strategy for inhibiting tumor progression and reprogramming the tumor cells to undergo cell death [69,70,71].

**DNMT inhibitors.** In human cells, DNA methylation is controlled by DNMTs, which include four members: DNMT1, DNMT3A, DNMT3B and DNMT3L. First, DNMT1 is required for maintaining the methyl group in the genome, then DNMT3A and DNMT3B are introducing de novo methyl groups catalyzed by DNMT3L [72]. Alterations in DNMTs are associated with tumors and disrupt the methylation pattern when abnormal function is triggered. Based on this principle, using DNMT inhibitor (DNMTi) genes, that are silenced due to hypermethylation, could be reactivated and inhibit tumor progression [73,74].

The use of DNMTi is a therapeutic strategy in several hematological malignancies, as their inhibition could be effective alone or in combination with chemotherapeutic drugs [75]. Patients diagnosed with Myelodysplastic Syndrome (MDS), Acute Myeloid Leukemia (AML) or Chronic Myeloid Leukemia (CML) have DNMTi in the standard of care strategies, and nowadays drug repositioning studies indicate that these therapies can be effective also in solid tumors [76].

In melanoma, few DNMTi were tested. Fludarabine is tested in a phase II clinical study (NCT00328861) or Disulfiram in another phase II clinical trial (NCT02101008) [76].

DNMTi are cytosine analogs which induce lower methylation in the DNA. The most common hypomethylating agents are nucleoside analogs and anti-sense DNA methyltransferase inhibitors. 5-Azacitidine, a nucleoside analog, is incorporated into DNA and RNA, while decitabine (5-aza-2′-deoxycytidine) in DNA inhibits proliferation [77].

DNA methylation and gene silencing were highlighted to be responsible for progression and relapse, as demonstrated in the case of MDS and AML patients who are not fit for induction chemotherapy or bone marrow transplant (BMT) [78]. Even though the mechanism of action in the case of myeloid malignancies is controversial, DNA hypomethylation may be responsible for the reactivation of certain genes that protect from cancer progression [79]. Moreover, the demethylating therapy should last for months to show visible results in myeloid malignancies, while in the case of solid tumors, until now, the use of DNMTi seems to lower resistance to checkpoint blockade and restore chemosensitivity to platinum-based drugs [80].

**Figure 3:**
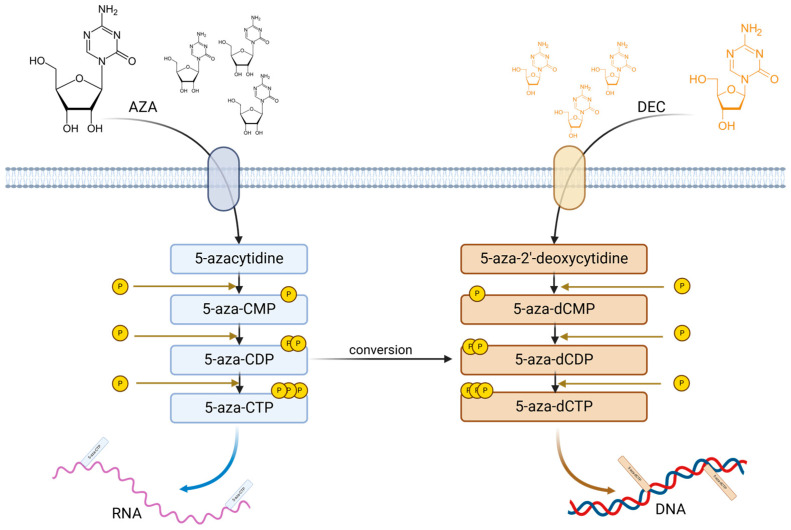
AZA and DEC phosphorylation and incorporation in DNA and RNA. Upon transportation into the cytosol, AZA and DEC undergo several phosphorylation steps; thus, AZA generates 5-aza-CMP, 5-aza-CDP and 5-aza-CTP, which are incorporated into RNA; DEC generates 5-aza-dCMP, 5-aza-dCDP and 5-aza-dCTP, which are incorporated into DNA. Moreover, 5-aza-CDP could be converted into 5-aza-dCDP, which, by further phosphorylation, it will generate 5-aza-dCTP.

**Hypomethylating agents.** The use of Azacitidine (AZA) and Decitabine (DEC) induces nucleic acid demethylation [81]. Both compounds are phosphorylated by different kinases and trisphosphate AZA and trisphosphate DEC are generated. Triphosphate AZA is incorporated in RNA, disrupting the protein synthesis and triggering apoptosis, while triphosphate DEC is incorporated into DNA promoting DNMT degradation by specific enzymes which leads to a loss of DNA methylation and to gene expression modifications. According to Diesch et al., AZA uptake is mediated via human concentrative nucleoside transporter and DEC through human equilibrative transporter; further, 1-uridine-cytidine kinase (UCK) and deoxycytidine kinase (DCK) promote 5-aza-CTP and 5-aza-dCTP generation. Part of 5-aza-CDP could be converted into dCDP variant shifting through DNA incorporation as DEC products. Both AZA and DEC incorporate into DNA and RNA inducing DNMT1 degradation and demethylation, reactivating silenced genes and modulating various biological processes (Figure 3) [82].

DNMT inhibitors are promising anticancer drugs, restoring the expression of tumor suppressor genes by reversing abnormal methylation patterns and also enhancing sensitivity to chemotherapy. While their melanoma applications are still under study, these agents might help resist the blockade of immune checkpoint inhibitors and enhance the immune response toward tumors. Properly optimizing treatment protocols and adequately comprehending the immunomodulatory characteristics of these agents will be instrumental in maximizing the observed effectiveness either as single-agent therapies or in combined schemes.

### 2.3. DNMT Inhibitors: Potential Role in Melanoma Therapy

Melanoma is well regarded as a unique model in cancer immunology because of its complex relationships with the immune system. Hence, a complicated interplay among genetic mutations, environmental factors, and immune responses is key to the responses of treatment. Thirty to forty percent of all melanoma subtypes show a strikingly high tumor mutational burden (TMB). Tumors with extremely high TMB typically respond better to immunotherapy, according to the studies [18].

Immune checkpoint inhibitors are commonly used in melanoma therapy, however, despite the positive results obtained using CTLA-4 and PD-1 inhibitors, some patients may develop resistance, due to immune escape mechanisms. The immune escape mechanism may be triggered by the modifications in the DNA methylation pattern, as in the case of melanoma, promoter methylation in the case of *PTEN*, *CDKN2A* or *ARF* was observed in several melanoma patients leading to poor prognostic and cell cycle dysregulation. On the other hand, gene methylation can promote resistance to T-cell-based therapies [83].

Epigenetics enhances melanoma resistance to the effects of immunotherapy by regulating the expression of proteins responsible for the resistance. Aleotti et al. discuss the essential roles of global erosion of DNA methylation additive to overall, and aberrantly elevated methylation of promoter CpG islands in tumorigenesis. Also, it details a list of different genes exhibiting hypermethylation and hypomethylation patterns associated with melanoma [84,85].

Yang et al. demonstrated that fat mass and obesity-associated protein (FTO) increase melanoma proliferation rate and inhibit the response to anti-PD-1 blockade immunotherapy, thus leading to therapy resistance [86]. As FTO is recognized as m6A demethylase with a role in RNA methylation, having key roles in cell function. In melanoma, FTO variations are associated with a high risk of tumor development and progression, without any association with increased body mass index [87]. Thus, the role of m6A eraser (FTO) in melanoma, and its use in restoring sensitivity to immune therapies is still under evaluation. However, it was demonstrated that FTO knockdown triggers the m6A hypermethylation and increases RNA decay via YTHDF2 (m6A reader), moreover, it sensitizes melanoma cells to IFN and anti-PD-1 therapies [86]. FTO gene modulation and RNA methylation changes can sustain a better response to therapy in the case of melanoma.

Currently, immune checkpoint inhibitors in melanoma face challenges due to resistance to therapy. Lim et al. suggest that the resistance mechanism involves a loss of wild-type antigen expression and intense dedifferentiation, together with an aberrant antigen presentation due to dysregulated MHC expression and nonetheless the immune cell exclusion due to PTEN loss; thus, restoring antigen expression and immunity stimulation, could reverse resistance to therapy [88].

As melanoma progresses from benign to malignant, cells may acquire cancer stem cell markers, enabling them to form metastatic sites; simultaneously, the loss of DNA hydroxy methylation is associated with the transition to malignancy and contributes to tumor progression [89]. In the EMT mechanism, the loss of E-cadherin is specific, and the matrix metalloproteases (MMPs) are upregulated to stimulate invasion and metastasis. The αVβ3 integrin becomes overexpressed and promotes melanoma expansion, via MMP2 expression. The epigenetic modulation of αVβ3 integrin might be involved in melanoma progression, furthermore, latexin (a negative regulator of HSCs) is downregulated in one-out-of-two melanomas, with its promoter region hypermethylated in melanomas and other cancers [34].

Currently, DNMT inhibitors have been tested in combination with other therapeutic agents and several studies have been presented by Hanly et al., highlighting their outcomes [90]. The use of Vemurafenib and decitabine, induced DNMT1 upregulation via the MAPK pathway, hypermethylating *BRAFV600E* mutant gene [91]. Decitabine was used in a combinatory therapy with an alkylating agent, Temozolomide, with an increased sensitivity to Temozolomide therapy as the main outcome [92]. Azacytidine has shown promising effects when combined with immune checkpoint inhibitors, such as anti-PD-1 antibody (pembrolizumab), by promoting PD-L1 expression, and with anti-CTLA-4 antibody, by enhancing tumor recognition and response by T-cells [93].

DNMT inhibitors show promising results in multidrug therapies in melanoma; thus, the use of DNMTi and immunotherapies could offer promising therapeutic alternatives modulating the epigenetic regulatory axis. The outcomes could reveal novel exploitable cancer immunotherapy targets in melanoma.

## 3. Histone Modifications in Melanoma

The structure of chromatin is based on histone proteins (H3, H4, H2A, H2B, H1), which compact eukaryotic DNA into nucleosomes [94]. Histone modifications cause structural changes at the chromatin level, influencing DNA processes such as transcription, replication, repair, and recombination. These modifications can influence gene expression and are fundamental to numerous biological processes. Key types of histone modifications include acetylation, methylation, and phosphorylation. Dysregulation of these processes can contribute to tumorigenesis by disrupting normal gene regulation and chromatin structure [95].

Histone acetylation is a dynamic process regulated by the action of two families of enzymes: histone acetyltransferases (HATs) and histone deacetylases (HDACs). HATs catalyze the transfer of acetyl groups to lysine residues, facilitating transcription. HDACs reverse acetylation, restoring the positive charge of lysine and acting as transcriptional repressors, stabilizing chromatin architecture [95].

In melanoma, deregulation of histone acetylation contributes to tumorigenesis by repressing tumor suppressor genes, such as *p14ARF* and *p16INK4a* [96], while hypoacetylation can cause downregulation of tumor suppressor genes involved in the PI3K/Akt pathway [94]. Melanoma treatment commonly involves inhibitors targeting the mitogen-activated protein kinase (MAPK) pathway; however, resistance frequently leads to relapse. In cancer, histone deacetylases (HDACs) are often overexpressed, and HDAC inhibitors can promote hyperacetylation of HSP90, leading to the degradation of c-RAF and Akt—key signaling molecules involved in tumor growth and resistance to MAPK inhibitors in melanoma [96].

These inhibitors may also be mediators of p53 activity contributing to therapeutic resistance and activating the NF-κB pathway, which regulates the production of cytokines, transcription of antiapoptotic proteins, and immune response [96]. Aberrant histone acetylation has been shown to affect tumor cells and tumor-infiltrating lymphocytes by regulating the TRAIL/Apo2L apoptotic pathway or proapoptotic proteins from the Bcl-2 family (Bim, Bax, and Bak) in melanoma, highlighting the therapeutic potential of HDAC inhibitors in melanoma [94].

Histone methylation occurs on lysine and arginine, modifying chromatin function without affecting histone charge. Lysine can be mono-, di-, or tri-methylated, influencing gene activation or repression, and the key enzymes involved are SUV39H1 (for H3K9 methylation) and SET7/9 (for H3K4 methylation). Demethylation is performed by enzymes such as LSD1 and proteins with the JmjC1 domain [95].

SETDB1 is an enzyme that methylates histone H3 on lysine 9 (H3K9), accelerating melanoma formation. SETDB1 overexpression is associated with aggressive tumor behavior, including lymph nodes and/or distant metastases. It has also been shown that the cytoplasmic expression of SETDB1 correlates significantly with a higher methylation frequency and suppressed expression of the p16 tumor suppressor [94].

EZH2 is the catalytic subunit of the H3K27 methyltransferase in the PRC2 complex, responsible for transcriptional repression by adding the H3K27me3 modification. In melanoma, EZH2 expression is associated with a high proliferation rate due to repressive H3K27 methylation, and its deficiency can have a proapoptotic effect by normalizing *CDKN1A* locus acetylation and eliminating histone deacetylases [94]. Additionally, EZH2 can interact with DNA methylases, creating a link between histone and DNA methylation. Activating mutations of EZH2 are identified in approximately 3% of melanomas, contributing to tumor progression. EZH2 inhibitors, including specific small molecules, can reduce cell growth and melanoma metastases, making EZH2 a potential therapeutic target [96].

JHDM1D (or KDM7A) is a histone demethylase that participates in the epigenetic regulation of genes by modifying the methylation level of histone. Increased expression of the histone demethylase JHDM1D under nutrient deprivation suppresses tumor growth by downregulating angiogenesis. Trimethylation of histone H3 lysine 79 and phosphorylation of H3 threonine 80 (H3K79me3T80ph) have been identified as markers for a subset of primary cutaneous melanomas with more aggressive clinical behavior [97].

Histone acetylation promotes tumorigenesis by enhancing YTHDF2 expression. YTHDF2 then recognizes m6A-modified PER1 and TP53 mRNAs and facilitates their degradation, accelerating ocular melanoma tumorigenesis [98].

Recent studies suggest that histone modifications could have significant diagnostic value. Immunohistochemical analyses performed on melanocytic tissues have shown H4K20me loss in 66% of malignant melanomas, compared to only 14% of benign nevi. Also, H3K27me3 levels were either completely lost or strongly expressed in 72% of malignant melanomas, suggesting that these PTMs (post-translational modifications of histones) could be useful biomarkers for melanoma diagnosis [99].

**Figure 4:**
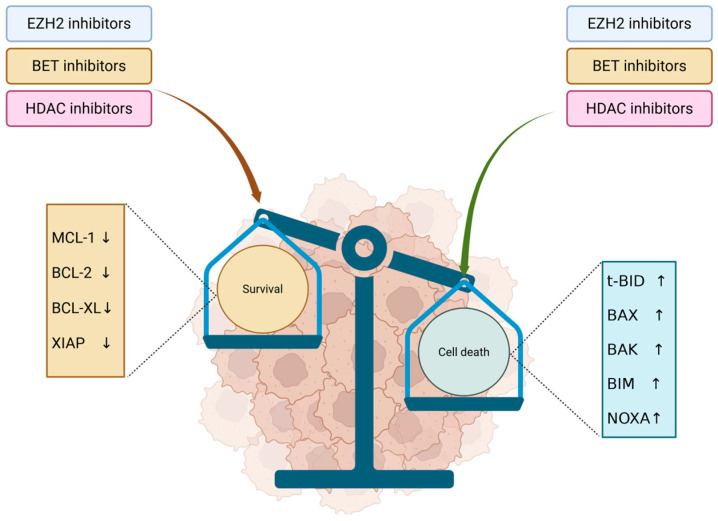
Antiapoptotic and proapoptotic signals modulated by HDACi, EZH2i and BETi. EZH2, BET and HDAC inhibitors lead to inhibition of antiapoptotic molecules, lowering the survival signaling in tumor cells. In parallel, they stimulate proapoptotic molecules, promoting cell death and inducing an overall antitumor effect. ↓—inhibition; ↑—stimulation.

The delicate balance between cell survival and death is tightly regulated by pro- and antiapoptotic signals. Histone modifiers play a crucial role in this process by downregulating antiapoptotic proteins and reactivating programmed cell death (Figure 4). Agents such as EZH2 inhibitors, HDAC inhibitors, and BET inhibitors can alter the histone acetylation and methylation landscape, thereby promoting apoptosis in cancer cells. Proapoptotic molecules such as t-BID, BAX, BIM, BAK or NOXA could be stimulated to sustain cell death, while antiapoptotic molecules such as MCL-1, BCL-2, BCL-XL or XIAP could be inhibited [96].

### 3.1. Histone Acetylation and HDAC Inhibitors

The balance between histone acetylation and deacetylation is regulating chromatin structure, with histone acetyltransferases that neutralize histone positive charging modulating the binding between histones and negatively charged DNA. Thus, acetylation opens the chromatin increasing gene transcription. On the other hand, the histone deacetylase compacts chromatin [100]. Histone acetylation is crucial in melanoma progression and many malignancies through HDAC and bromodomain and extra terminal protein BET. HDACs modulate gene expression, including genes that encode PD-1/PD-L1, influencing immune evasion. Therefore, studies have demonstrated that HDACi may reduce the expression of PD-L1 and sustain MHC class I expression, modulating immunity and immune responses. On the other hand, BET protein inhibition induced an antiproliferative effect and inhibited tumor progression through cell cycle arrest in G1 [101].

HDACi represents a promising avenue for melanoma therapy, targeting HDACs that modulate chromatin and influence gene expression. Histone hyperacetylation induces cell cycle dysregulation, triggers apoptosis, and modulates angiogenesis and immune responses. Several HDACi have been tested in melanoma, including short-chain fatty acids like valproic acid (VPA) or pivaloyloxymethyl butyrate (AN-9). VPA-induced DNA damage and cytotoxicity in melanoma cells combined with chemotherapy and immunotherapy showed limited clinical benefits. AN-9 has antimetastatic potential through angiogenesis inhibition, with concerns related to side effects such as hyperglycemia and fatigue [102]. Benzamide-based HDACi like entinostat showed high selectivity to class I HDACs with a well-tolerated dose of 6 mg/m^2^ and increased antitumor activity. The acetylation of histones 3 and 4 was significantly increased, but the side effects like neurotoxicity and hepatotoxicity still need attention [103].

Together with the DNA and RNA methylation landscape, the balance between acetylation and deacetylation is controlling the transcription switch through HDAC and HAT activity. Acetylated histones are open to transcription, while the lack of acetyl groups closes chromatin and inhibits transcription. Reprogramming HDACs and BETs could offer new perspectives on melanoma therapy and other malignancies.

### 3.2. Histone Methylation and EZH2 Inhibitors

Histone methylation refers to the chemical alteration of amino acids that are constituents of histone proteins, through the binding of either one (mono), two (di), or three (tri) methyl groups. This process is primarily associated with repression of transcription, or silencing; however, the methylation of certain lysine and arginine residues in histones can activate transcription. Notable residues for lysine methylation studies have primarily focused on the methylation of lysine residues at K4, K9, and K27. Lysines can exist in a mono-, di-, or tri-methylated form. Trimethylation of lysine 4 on histone H3 (H3K4me3) indicates a completely activated promoter associated with actively transcribed genes. The other possibility of demethylation at lysine 4 (H3K4me2) can exist in the inactive or actively expressed euchromatin genes. As a negative regulator of the H3K4 mark, H3K9 demethylation (H3K9me2) signifies gene silencing in euchromatin, which is important for proliferating cells. The mark of H3K9me3 is found in “gene-poor” pericentric heterochromatin. Methylation of lysine 27 on histone H3 (H3K27me) is typically an issuer of transcriptional repression during various developmental processes [104,105].

Different functional outcomes can be displayed depending on the histone methylation degree, which is a dynamic process which involves histone-modifying enzymes that are recruited at specific DNA sequences, marks on histone tails, DNA methylation patterns or even noncoding RNAs. Methylation marks can be altered and may modulate the phenotype of the cells, and in certain conditions could play a critical role in disease development, including cancers. Histone methylation is crucial for DNA repair, cell cycle, transcription, aging and differentiation; thus, the landscape of histone methylation could represent a target for different targeted therapies in different malignancies [106].

EZH2 is a catalytic subunit of Polycomb repressive complex 2 (PRC2), a histone methyltransferase, that is well conserved and targets lysine-27 of H3. If methylated, H3-K27 is associated with different gene silencing in various organisms, while in humans, EZH2 is overexpressed in tumors and its functions are altered. In specific cases, EZH2 overexpression was linked to an advanced stage of the disease and poor prognosis [107].

In the context of drug-resistant tumors and immune evasion mechanisms, a recent study completed by Yu et al. suggested that EZH2 inhibitors modulate epigenetic levels, restoring gene function in aberrant cells. In this case, it was shown that capsanthin, a natural compound, can inhibit PD-L1 expression which resulted in reduced cancerous cell migration in triple-negative breast cancer brain metastases [108].

In another study, Cui et al. demonstrated that Mi-2β, a remodeling factor of chromatin, activates the methylation of EZH2, promoting immune evasion in melanoma [109]. As Mi-2β regulates the adaptive immune response, its silencing can induce an immune response to anti-PD-1 therapy in melanoma [109].

The most studied EZH2 inhibitor in melanoma is Tazemetostat. This is a highly efficient drug due to its capability to maintain T-cell stemness, resulting in efficient prevention of T-cell exhaustion, which in turn allows for a stronger immune response against melanoma cells [110].

Epigenetic modifications serve as important regulators of gene expression, consequently impacting cancer progression and treatment responses. Of the epigenetic regulators of gene expression, histone methylation represents one of the most important features affected by histone-modifying enzymes, such as LSD1, DOT1L, and EZH2. The expression of these enzymes is increased in malignancies and is considered promising new therapeutic targets in oncology. LSD1, a histone demethylase, modifies chromatin structure by removing methyl marks from histone H3 and regulating the expression of localized genes [111]. Cell proliferation and increased tumorigenicity are related to overexpression of LSD1, particularly in lung, breast, and colorectal tumors. LSD1 inhibition treatment disrupts these tumorigenic processes, especially in small-cell lung cancer, where LSD1 inhibition affects the differentiated state of tumors [112]. Similarly, DOT1L, a histone methyltransferase, plays a significant role in methylating histone third lysine 79 (H3K79), modulating transcriptional regulations and functions in cell differentiation. Mutations in DOT1L are implicated in Mixed lineage leukemia 1 rearranged AML, making DOT1L a significant contributor towards chemotherapy resistance. DOT1L inhibitors had exhibited promise in preclinical models of AML, but ultimately the clinical applications of DOT1L inhibitor treatments were restricted due to poor responses which signal the potential benefit of multi-arresting malignant processes through combination treatments [111].

Taken together, LSD1, DOT1L, and EZH2 inhibitors have the potential to induce substantial changes in histone methylation, thereby significantly altering the epigenetic landscape of cancer cells. Further research is needed to explore their synergistic potential in combination therapies, with the goal of achieving more effective and targeted anticancer outcomes.

## 4. TET Enzymes and Their Role in Melanoma

TET proteins are a family of dioxygenases involved in DNA demethylation. They act by oxidizing 5-methylcytosine (5mC) to 5-hydroxymethylcytosine (5-hmC), 5-formylcytosine (5-fmC)and 5-carboxycytosine (5-caC) in a Fe(II)/alpha-ketoglutarate-dependent manner [113]. 5hmC is involved in transcriptional regulation in normal development and cancer [114]. Normal melanocytes that express regular levels of TET enzymes were found to have a high 5-hmC content, shaping the epigenetic landscape of normal cells. On the other hand, when normal melanocytes gain a malignant phenotype associated with melanoma, they suffer a decrease in the levels of TET enzymes, especially TET2, leading to a genome-wide loss of 5-hmC. Therefore, the loss of TET enzymes and the subsequent epigenetic reshaping were determined to be a hallmark of melanoma progression and identification [44]. It was also found that the loss of Tet2 acts in a cooperative manner with mutant NRas, driving melanoma genesis and accelerating melanoma progression. Moreover, Tet2-deficient tumors developed much earlier than Tet2+/+ tumors [115].

The epithelial-to-mesenchymal transition (EMT), a key feature of melanoma progression, is a process through which cells originating in the epithelium gain a mesenchymal phenotype, becoming more invasive and migratory [116]. TET enzymes had lower expression in cultured melanoma cells that underwent TGF-β1-induced epithelial-mesenchymal transition (EMT). Additionally, the overexpression of TET2 in these melanoma cells effectively reversed the TGF-β1-induced EMT in vitro and inhibited tumor growth in vivo [117].

TET enzymes, particularly TET2, have been implicated in melanoma immune evasion and resistance to immune checkpoint inhibitors. Research indicates that TET2 plays a role in activating IFN-γ-induced chemokine and PD-L1 gene expression by mediating the IFN-γ/JAK2/STAT1 signaling pathway, thereby contributing to an immunosuppressive tumor microenvironment. Therefore, the Tet2 loss in a murine melanoma cell line conferred resistance to antitumor immunity mediated by CD8+ T-cells and immunotherapy [118]. Moreover, the same authors demonstrated that TET2 enhances the expression of specific MHC class I antigen presentation genes, including TAP1 and TAPBP, in a murine melanoma cell line and other cancer cell lines. Tet2 knockout in melanoma cells resulted in reduced antigen presentation and impaired activation of CD8+ T-cells, underscoring its role in tumor immune recognition [119].

TET2 downregulation and low levels of 5-hmC represent hallmarks for melanoma progression, while another marker of aggressive malignant phenotype in melanoma is represented by the accumulation of the Hypoxia-Inducible Factor-1a (HIF-1a) [120].

The low levels of 5-hmC and the overexpression of HIF-1a in high-grade melanoma might suggest a correlation between TET enzymes and HIF-1a in the development of this malignancy. It was demonstrated that HIF-1a silencing in a metastatic melanoma cell line led to a significant increase in the amount of TET2 enzyme, at both mRNA and protein levels. Therefore, HIF-1a seems to regulate the expression of the TET2 enzyme, contributing to the modified epigenetic landscape of melanoma and it might represent a future target for melanoma therapies [121].

Mutations of the TET encoding genes frequently occur in different malignancies, being well characterized in hematological malignancies. Although mutations in TET genes are relatively rare, their catalytic activity may be dysregulated due to alterations in the methylation landscape, which can impact their function and contribute to tumor progression [122].

**Figure 5:**
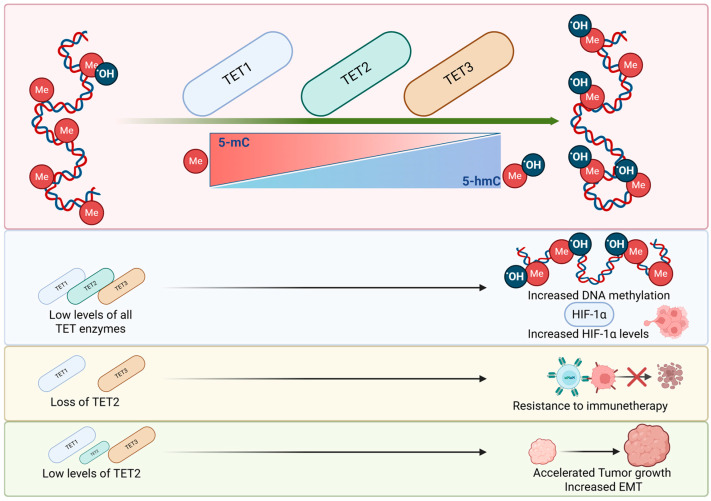
The impact of TET enzymes on tumorigenesis. TET1, TET2 and TET3 control the conversion of 5-mC into 5-hmC initiating the demethylation process. Low levels of TET enzymes are related to increased DNA methylation and increased levels of HIF-1α. Low levels of TET2 could accelerate tumor growth and EMT. While the loss of TET2 could lead to resistance to immunotherapies.

TET enzymes are critical for maintaining the balance between 5-mC and 5-hmC. As shown in Figure 5, TET dysregulation could lead to an accumulation of 5-mC increasing the DNA methylation and stimulating tumor growth. Furthermore, low levels of TET, are associated with low 5-hmC and increased HIF1α activity, promoting angiogenesis. On the other hand, increased TET2 levels can protect cells from EMT and resistance to therapy, highlighting the importance of TET enzymes in tumor development.

### 4.1. TET Enzymes and DNA Demethylation

TET enzymes oxidize 5mC generating intermediate products until they clear cytosine, modulating DNA methylation. Studies show that TET1 deletion can cause a drastic decrease in 5hmC, impairing stem cell identity at the embryonic level [123]. Furthermore, changes in methylation status due to TET1 inactivity or impaired function could induce changes in epithelial cells, liver damage and demyelination [124].

TET1 controls iron homeostasis through *RNF217* promoter demethylation; it promotes pluripotent stem cell induction, and its abnormal expression is associated with different diseases [113]. Aberrant methylation due to loss of TET1 may be responsible for several malignancies, while low levels of TET1 were identified in several non-malignant diseases like polycystic ovary syndrome and hypertension [125].

TET2 mutations are worsening overall survival in patients suffering from AML [126]; however, other studies have shown no direct involvement in the survival of AML patients [127]. Contradictory studies were reported and highlighted by Zhang et al., where TET2 was evaluated for its oncogenic activity and for its protective activity in different malignancies, depending on which promoter regions were influenced by changes in methylation landscape [113].

From embryogenesis to adult tissues, TET3 is involved in demethylation, while its low expression induced changes in growth rate, and neuronal activity and even induced behavior changes [128]. Studies link TET3 abnormalities to diabetes, as maintained hypermethylation on genes that control insulin secretion induces changes in glucose metabolism [129].

Collectively, TET enzymes are orchestrating the DNA demethylation steps, modulating each other’s function, being involved in maintaining cell identity, epigenetic reprogramming, and controlling DNA methylation patterns [113].

The cooperation and compensation of TET enzymes were interestingly demonstrated in mice models, where individual knockout of TET1 and TET2 did not induce death, while double knockout resulted in mice death, showing the connection between TET enzymes and highlighting the fact that the lack of one TET enzyme could be compensated by the other TET enzymes. On the other hand, TET3 deletion showed a unique effect by inducing death in the neonatal state, indicating its critical role in development, without a certain mechanism established [130].

DNA methylation and demethylation are actively regulated and have major roles in biological processes such as epigenetic memory, genomic imprinting and development, and changes in the methylation landscape can often lead to multiple diseases, including autoimmune diseases and cancers. Moreover, hypermethylation patterns are typically associated with gene silencing. Therefore, the development of epigenetic editing tools allows for the alteration of specific regions of the genome, to study the effects of epigenetic changes, or to selectively silence/activate one or more genes in a specific context.

### 4.2. TET Modulators

As previously described, TET enzymes are crucial for maintaining the balance between methylated and demethylated DNA. Dysregulations in TET enzyme expression and function could lead to different diseases and malignancies.

TET modulation could be initiated through different therapies that act as activators and inhibitors, which may be newly developed drugs or ones already in use that are repositioned [131].

A study focused on MDS and AML highlighted the fact that vitamin C targeted all TET enzymes and induced an increase in 5hmC concentration, leading to a DNA hypomethylation which was favorable in inhibiting tumor development [132]. In MDS, SIRT1 agonist SRT1720 targeted TET2 and enhanced its activity disrupting MDS maintenance [133].

Wu et al. evaluated the link between AMPK and TET2 linking diabetes to cancer, by showing that DNA methylation is affected in hyperglycemic conditions. TET2 destabilization was induced by AMPK-mediated phosphorylation at Ser99 and the tumor suppressive function of TET2 was dysregulated. The use of metformin stabilized TET2 and restored 5hmC levels after rescuing Ser99 phosphorylation, offering a new perspective on using drug repositioning in epigenetic reprogramming for cancer treatment [134].

TET inhibitors like 2HG, fumarate, succinate, Bobcat339, TETi76, C35 itaconate and others were used to target TET enzymes and to downregulate 5hmC levels in hematological malignancies, to induce hypermethylation in solid tumors and now synthetic compounds undergo in vitro and in vivo evaluation to determine their potential as TET inhibitors in various malignancies [131].

2HG induces DNA hypermethylation by targeting TET2, mediating gene silencing and tumor progression in hematological malignancies. 2-Hydroxyglutarate (2HG) competes with α-ketoglutarate for α-ketoglutarate-dependent dioxygenases, which include histone demethylases and the TET family of methylcytosines dioxygenases. Inhibition of these enzymes results in epigenetic changes and blocks cellular differentiation [135].

Fumarate and succinate, two Krebs cycle products, are competitors of αKG-dependent dioxygenases like histone demethylases and the TET family. High levels of these two products downregulate 5hmC levels and induce DNA hypermethylation, through TET inhibition [136]. Chen et al. revealed that Itaconate can inhibit TET2, and, in in vivo models, 5hmC was downregulated and reduced LPS-induced liver and lung injury. This indicates that Itaconate can play an immunomodulatory role, due to its selective inhibition of TET enzymes [137].

Zhang et al. evaluated Dimethyloxallyl glycine (DMOG) as a Tet3 inhibitor in solid tumors. MCF-7 cells showed aberrant DNMTs and TETs which may be responsible for the low methylation of human leukocyte antigen-G promoter gene region, leading to tumor progression. The use of DMOG shows promising results, as the compound could restore DNA methylation and inhibit MCF-7 proliferation. However, further studies need to confirm these findings [138].

TET2 inhibition could represent a promising approach to reactivate immune cells from the TME. TET1 was negatively regulated by p65 in different solid tumors including melanoma, suggesting that targeting this mechanism could restore the response to therapy and may reduce the metastatic potential of tumor cells [139].

Together with DNMTs and histones, TET enzymes modulate DNA methylation and transcription through complex and interconnected processes. Dysfunctions in one of these processes might be compensated by other mechanisms, while in certain conditions, when the compensation mechanisms cannot be activated, the methylation landscape in DNA and histones could suffer severe changes that could trigger or silence different genes. The resulting gene modulations could initiate tumor development, could induce resistance to therapy and may disrupt the normal function of different cells and tissues.

## 5. Conclusions and Perspectives

Epigenetic therapies are a newly emerging and exciting option in the treatment of melanoma, with the goal of reducing systemic toxicities and addressing patient symptoms through selective mechanisms that target gene expression. An integrative approach to studying melanoma phenotypes, genotypes, and epigenetics would be helpful to better understand the variance of clinical signs of the disease and address treatments that are potentially more efficacious. The use of epigenetic-based therapy is still early, with a growing understanding of its efficacy. Future considerations may include the use of epigenetic agents alone, in combination with similar epigenetic modifiers, or traditional chemotherapies. More research needs to be conducted to develop new integrative approaches to improving the treatment of melanoma.
